# P-1244. Optimizing Dosing of Penicillin in Combination with Ceftriaxone Against *Enterococcus faecalis*: An *in vitro* Pharmacodynamic Model

**DOI:** 10.1093/ofid/ofae631.1426

**Published:** 2025-01-29

**Authors:** Olivia G Funk, Nadine Abulmagd, Jaclyn A Cusumano

**Affiliations:** Long Island University, Brooklyn, New York; Long Island University, Brooklyn, New York; Long Island University, Brooklyn, New York

## Abstract

**Background:**

Penicillin may replace ampicillin in combination with ceftriaxone against *Enterococcus faecalis* endocarditis due to ampicillin stability, but optimal penicillin dosing is unknown. Penicillin pharmacodynamic (PD) optimization is especially important against borderline-penicillin-resistant, ampicillin-susceptible *E. faecalis* isolates (“borderline-PRASEF”; MIC 4-8 µg/mL; CLSI breakpoint ≤8 µg/mL) where, *in vitro*, ampicillin/penicillin-ceftriaxone has shown less activity.

Table 1

**Figure:**
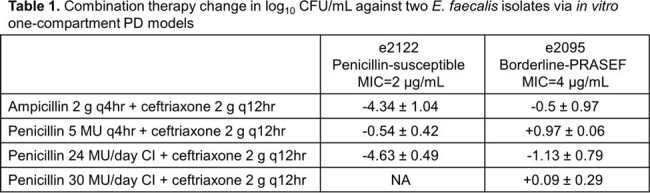

Combination therapy change in log10 CFU/mL against two E. faecalis isolates via in vitro one-compartment PD models

**Methods:**

Penicillin intermittent infusion was compared to continuous infusion (CI) alone and with ceftriaxone against a penicillin-susceptible (e2122; MICs [µg/mL]: ampicillin 1, penicillin 2, ceftriaxone 256) and a borderline-PRASEF (e2095; MICs [µg/mL]: ampicillin 1, penicillin 4, ceftriaxone 2048) isolate via 48-hour *in vitro* one-compartment PD models, in duplicate, with a starting inoculum of 10^6^ CFU/mL. The max penicillin intermittent dose, 5 MU every 4 hours (*f*T>MIC: e2122, 53.1%; e2095, 40.7%) and most utilized CI dose, 24 MU/day (*f*T>MIC: e2122 and e2095, 100.0%) were tested. Ampicillin 2 g every 4 hours (*f*T>MIC: e2122 and e2095, 100.0%) alone and with ceftriaxone 2 g every 12 hours was used as a comparator. Therapeutic enhancement was ≥2-log_10_ CFU/mL decrease from the most active single agent. Adequate activity was a ≥2-log_10_ CFU/mL decrease from starting inoculum of 10^6^ CFU/mL.

**Results:**

No antibiotic alone achieved adequate activity. Against e2122, when combined with ceftriaxone, ampicillin and penicillin CI 24 MU/day achieved adequate activity and therapeutic enhancement whereas penicillin 5 MU intermittent infusion plus ceftriaxone did not. Against e2095, no combination achieved adequate activity or therapeutic enhancement. Due to insufficient activity, we tested e2095 against the highest penicillin CI dose of 30 MU/day plus ceftriaxone which did not achieve either outcome.

**Conclusion:**

Optimizing penicillin *f*T>MIC via CI dosing is needed to achieve similar antibacterial kill to ampicillin-ceftriaxone against penicillin-susceptible *E. faecalis*. Conversely, even the max penicillin dose of 30 MU/day (*f*T>MIC 100.0%) as well as standard of care ampicillin-ceftriaxone was unable to maintain activity against borderline-PRASEF.

**Disclosures:**

**Jaclyn A. Cusumano, PharmD, BCIDP**, Basilea Pharmaceutica: Grant/Research Support

